# Correspondence

**Published:** 1876-09

**Authors:** 


### Correspondence.

### HOMŒOPATHY IN THE UNIVERSITY OF MICHIGAN.

The following correspondence is self-explanatory:

UNIVERSITY OF MICHIGAN,
ANN ARBOR, June 16, 1876.

*Rush Medical College, Chicago, Ill.:*

TO THE DEAN. DEAR SIR — I am instructed by our Faculty to inquire whether your College will accept the tickets, and recognize the diploma of the Department of Medicine and Surgery, (the old regular school) of this University?

Frequent inquiries are made by our students as to whether their time will be allowed in other colleges, and we desire now to be able to state officially to them what the position of the leading medical colleges of the country is on this question. An early reply will oblige,

Yours, very truly,
E. S. DUNSTER, M.D., *Acting Dean.*

RUSH MEDICAL COLLEGE,
CHICAGO, July 5, 1876.

The following resolution, adopted to-day by the Faculty of this College, contains an answer to questions relative to Homœopathy sent to this institution by the Dean of the University of Michigan:

"*Resolved*, That the time and attendance of students upon lectures of the medical department of the University of Michigan, up to and including the last regular session of that College, may be recognized as part of the requisites for graduation in this College; but such time and attendance shall not hereafter be accepted so long as the teaching of Homœopathy, in whole or in part, shall be included in the course of study at that institution."

J. H. ETHERIDGE, *Assistant Secretary.*

UNIVERSITY OF MICHIGAN,
ANN ARBOR, Mich., July 10, 1876.

J. H. ETHERIDGE, M.D.,
*Sec'y Rush Medical College, Chicago.*

DEAR SIR — Your favor of the 5th inst. is just received. In reply, I beg leave to ask again for more definite information on the point in question. The last clause of your resolution says, "so long as the teaching of Homœopathy, in whole or in part, shall be included in the course of study *at that institution.*" Homœopathy is taught in the "College of Homœopathy," but not in any way direct or indirect, in the "Department of Medicine and Surgery," which is the old medical school of this University. Our school and our students have no more association or relation with Homœopathy than your school and your students. The Homœopathic students, however, are required to listen to our lectures in the fundamental branches of medicine. The curriculum is unchanged, save that it is gradually being increased in severity of requirements, both for admission and for graduation. Will you therefore please advise me whether, by the words, "*at that institution,*" in your resolution, you mean the "Department of Medicine and Surgery," *i. e.*, the old medical school, or whether you mean the "University of Michigan," as a whole, which includes of course all its separate colleges, the Homœopathic among the rest. There has been, and still is, much misunderstanding of the situation in the University of Michigan relative to Homœopathy, and the wording of your resolution leads me to infer that you have adopted the opinion which has been very industriously circulated by our enemies, that Homœopathy is taught in our College, and that the Homœopathic professors are part of our faculty; in both of which statements there is not a grain of truth. Regretting to again trouble you with this matter, and hoping for a speedy reply,

I am very respectfully your servant,
E. S. DUNSTER, M.D., *Acting Dean.*

RUSH MEDICAL COLLEGE,
CHICAGO, July 17, 1876.

PROF. E. S. DUNSTER, *Acting Dean:*

MY DEAR SIR -- Your note to the Assistant Secretary, relative to the clause, "at that institution," which is contained in the resolution adopted by the Faculty of Rush Medical College, has been handed over to me for reply. In response to your desire for "more definite information," I have to say:

*First*, That the legislative device of the Board of Regents of the University of Michigan fails entirely to obscure the fact that the Medical Department, in connection with two Homœopathic professors, is engaged in educating Homœopathic students to become Homœopathic practitioners.

*Second*, That when that device calls two Homœopathic professors a "Medical College," or a "College of Homœopathy," and requires the Medical Department to instruct the Homœopathic students on all subjects except Homœopathic therapeutics and practice, it practically establishes a copartnership of effort in the education and manufacture of Homœopathic doctors.

*Third*, That when that device requires the Medical Department not only to instruct Homœopathic students, but also to report to the Board of Regents upon the proficiency of those students; and when, upon that report, and a similar report from the two Homæopathic professors, the board proceeds to confer upon those Homœopathic students the degree of M.D., the enactment of that device essentially marries the Medical Department to the so-called Homœopathic College, and degrades it to the general level of fraud, delusion, and farce.

I have the honor to be respectfully and sorrowfully yours,

MOSES GUNN.

UNIVERSITY OF MICHIGAN,
ANN ARBOR, Mich., July 19, 1876.

PROF. MOSES GUNN, M.D.:

*Dear Sir* — I have the honor to acknowledge the receipt of your communication of the 17th inst., and in reply beg leave to intimate that it does not answer the question which I submitted to the Assistant Secretary of your College. Instead thereof you give me your opinion of the situation here, which is no doubt very valuable and instructive, but which was entirely unsolicited, and is in no way pertinent to my query. I shall esteem it a favor if you will inform me what is meant in the resolution of your faculty by the words, "in that institution." Or, to put the question even more definitely, do you, by these words, refer to the old regular Medical College of this University, or to the new Homœopathic Medical College?

I have the honor to remain very respectfully and cheerfully, your obedient servant.

E. S. DUNSTER, M. D.,

*Acting Dean Dep't Med. and Surg. Univ. Mich.*

RUSH MEDICAL COLLEGE,
CHICAGO, July 20, 1876.

PROF. E. S. DUNSTER, *Acting Dean:*

MY DEAR SIR — If you are still in doubt as to the opinion of Rush Medical College, viz., that your Medical Department and two Homœopathic professors are educating and making Homœopathic doctors, and that thus, *in effect*, Homœopathy is taught by the "old" and *formerly* "regular" Medical Department of the University of Michigan, we must leave you to the comforts of your doubt as to our meaning in the term, "at that institution."

Respectfully and still sorrowingly yours,
MOSES GUNN.

